# TRAF4 positively regulates the osteogenic differentiation of mesenchymal stem cells by acting as an E3 ubiquitin ligase to degrade Smurf2

**DOI:** 10.1038/s41418-019-0328-3

**Published:** 2019-05-10

**Authors:** Jinteng Li, Peng Wang, Zhongyu Xie, Shan Wang, Shuizhong Cen, Ming Li, Wenjie Liu, Su’an Tang, Guiwen Ye, Guan Zheng, Hongjun Su, Mengjun Ma, Xiaohua Wu, Yanfeng Wu, Huiyong Shen

**Affiliations:** 1 Department of Orthopedics, The Eighth Affiliated Hospital of Sun Yat-Sen University, Shenzhen, P.R. China; 2 Department of Orthopedics, Sun Yat-Sen Memorial Hospital of Sun Yat-Sen University, Guangzhou, P.R. China; 3 Center for Biotherapy, Sun Yat-Sen Memorial Hospital, Sun Yat-Sen University, Guangzhou, P.R. China

**Keywords:** Stem-cell research, Ubiquitylation

## Abstract

TNF receptor-associated factor 4 (TRAF4), a member of the TRAF family, plays an important role in the embryogenesis and development of the bone system. Mesenchymal stem cells (MSCs), which are the primary origin of osteoblasts in vivo, are key cells in bone development; however, whether TRAF4 modulates the osteogenic capacity of MSCs has never been explored. In this study, we demonstrated that TRAF4 positively regulates the osteogenic process of MSCs both in vitro and in vivo. In addition, we further demonstrated that TRAF4 modulates the osteogenic process of MSCs by acting as an E3 ubiquitin ligase to mediate the K48-linked ubiquitination of Smurf2 at the K119 site and cause degradation. Furthermore, TRAF4 was abnormally decreased in bone sections of ovariectomized rat and osteoporosis patients. Taken together, our findings suggest that TRAF4 positively regulates the osteogenic differentiation of MSCs by acting as an E3 ubiquitin ligase to degrade Smurf2. These results emphasize the critical role of TRAF4 in bone formation and could not only improve the clinical use of MSCs in tissue engineering but also clarify the pathogenesis of bone metabolism disorders.

## Introduction

Mesenchymal stem cells (MSCs) are multipotent progenitor cells that can differentiate into osteoblasts, chondrocytes, and adipocytes [1, 2]. Due to their strong potential in osteogenic differentiation capacity, MSCs are considered to be the most promising cell types used in tissue engineering technology for bone regeneration and repair [3–5]. However, the molecular mechanism that governs the osteogenic differentiation of MSCs remains largely unknown and hampers the further development of MSC-based cell therapies for bone defects in the clinic. Therefore, to efficiently harness MSCs for therapeutic purposes, it is necessary to understand the molecular mechanisms underlying MSC osteogenic differentiation.

TNF receptor-associated factor 4 (TRAF4) is a unique member of the TRAF family [6]. Previous in vivo studies have clearly demonstrated that TRAF4 is involved in embryogenesis and that TRAF4 deficiency results in severe malformation of the skeleton system, which indicates that TRAF4 is indispensable in bone development [7]. However, further studies illustrating the concrete role of TRAF4 in the process of bone remodeling at the molecular biological level have not been reported. Osteoblasts are among the most important cells in regulating the bone remodeling process in vivo [8, 9], and MSCs are the major origin of osteoblasts [10, 11]. However, whether TRAF4 modulates the osteogenic capacity of MSCs has never been explored.

Smad ubiquitination-related factor 2 (Smurf2) is a member of the Hect domain family of E3 ubiquitin ligases [12, 13], and it interacts with and degrades various essential osteogenesis-related molecules including Smad1 and Runx2 to negatively regulate the osteogenic differentiation process [12, 14, 15]. Although the mechanism of Smurf2 in modulating the osteogenic differentiation process has been widely studied, the upper regulatory network to modulate the expression of Smurf2 during the osteogenic differentiation process of MSCs remains unclear.

Taken together, our findings suggest that TRAF4 positively regulates the osteogenic process of MSCs both in vitro and in vivo, can be a promising target not only to improve the efficiency of MSC-based tissue engineering therapy for bone regeneration and repair but also to treat the pathogenesis of bone metabolism disorders, such as osteoporosis.

## Methods and materials

### Isolation and culture of MSCs

This study was approved by the ethics committee of Sun Yat-Sen Memorial Hospital, Sun Yat-Sen University, Guangzhou, China. Written informed consent was obtained from all subjects included in the study. MSCs were isolated and cultured as previously described [16–18]. Briefly, MSCs were isolated and purified from bone marrow using density gradient centrifugation at 12,000 rpm for 30 min. The MSCs were then resuspended in Dulbecco’s modified Eagle’s medium (DMEM, glucose 1000 mg/L, GIBCO) containing 10% fetal bovine serum (FBS, Hangzhou Sijiqing Biological Engineering Material Company, Limited), seeded in flasks, and cultured in incubators at 37 °C and 5% CO_2_. After 48 h, the culture medium was replaced to remove nonadherent cells. The medium was replaced every 3 days thereafter. When the cells reached 80–90% confluence, the MSCs were digested using 0.25% trypsin containing 0.53 mM ethylenediamine tetraacetic acid (EDTA) and reseeded in new flasks as passage 1. The MSCs were expanded and used for subsequent experiments at passages 3–5.

### Culture of 293T cells

293T (human embryonic kidney cell line) cells were cultured in glucose DMEM (glucose 4500 mg/L, GIBCO) with 10% FBS at 37 °C under 5% CO_2_. When the cells reached 80–90% confluence, 293T cells were digested with 0.25% trypsin containing 0.53 mM EDTA and reseeded in new flasks.

### Osteogenic differentiation induction

MSCs were separately seeded in 12-well plates at a density of 0.5 × 10^5^ cells/well in culture medium as described above [16]. After 12 h, when cells were adherent to the wells, the culture medium of the MSCs was changed with osteogenic differentiation medium (OM), consisting of DMEM (glucose 1000 mg/L) with 10% FBS, 100 IU/mL penicillin, 100 IU/mL streptomycin, 0.1 μM dexamethasone, 10 mM β-glycerol phosphate, and 50 μM ascorbic acid (Sigma-Aldrich). The medium was changed every 3 days.

### Alizarin red S (ARS) staining and quantification

MSCs are believed to accumulate Ca^2+^ and inorganic phosphate during osteogenesis, which serve as nucleating agents for the formation of hydroxyapatite (Ca_10_(PO_4_)6(OH_2_)2), the primary inorganic component of bone [19, 20]. Alizarin red S can react with calcium ions to form orange-red complexes that can be directly observed by the naked eye or with a microscope [19]. Thus, Alizarin red S staining can be used to quantify the amount of extracellular mineralized matrix, which is a long-term cumulative effect of osteogenesis [19].

MSCs were first fixed in 4% paraformaldehyde for 30 min and then stained with 1% ARS (pH 4.3) for 15 min at room temperature. To remove nonspecific staining, the stained cells were then washed at least three times with phosphate-buffered saline (PBS). Subsequently, the stained cells were observed under a microscope and photographed. For ARS quantification, 10% cetylpyridinium chloride monohydrate (Sigma-Aldrich) was used to destain the cells for 1 h at room temperature. Thereafter, 200 μL of the liquid was transferred to a 96-well plate, and the spectrophotometric absorbance was measured at 562 nm.

### ALP staining and activity assay

Alkaline phosphatase (ALP) is a membrane-bound exoenzyme that has been implicated in bone formation and mineralization [21], and is a key marker for monitoring the early stages of osteogenic differentiation [21, 22]. Since the expression of ALP can be easily qualitatively assessed by ALP staining and quantitatively assayed through ALP activity assays, the evaluation of ALP is another typical means of monitoring osteogenesis.

For ALP staining, the differentiated cells were fixed in a formaldehyde solution and stained using a BCIP/NBT alkaline phosphatase kit (Beyotime Institute of Biotechnology) in accordance with the manufacturer’s instructions. Stained cells were photographed.

For the ALP activity assay, ALP activity kits (Nanjing Jiancheng Biotech) were used. Briefly, MSCs were lysed using RIPA buffer containing protease and phosphatase inhibitors (Beyotime Institute of Biotechnology, China). Lysates were centrifuged at 14,000 rpm at 4 °C for 30 min, and the supernatants were incubated with reaction buffer at 37 °C for 15 min. After adding stop solution, the absorbance was measured at 405 nm. A Pierce bicinchoninic acid (BCA) protein assay kit (Thermo Fisher Scientific) was used to detect the total protein concentration. ALP activity was normalized to the total protein content at the end of the experiment and reported as units per gram of protein per 15 min (U/gpro/15 min).

### Western blot analysis

The western blot protocols were reported previously [16]. Briefly, cells were washed three times with ice-cold PBS. Then, the cells were harvested and lysed in RIPA buffer containing protease and phosphatase inhibitors for 30 min on ice. Lysates were obtained via centrifugation at 14,000 rpm at 4 °C for 30 min. A Pierce BCA protein assay kit (Thermo Fisher Scientific) was used to detect the total protein concentration. Equal amounts of each sample diluted in 5× sodium dodecyl sulfate (SDS) loading buffer were subjected to SDS–polyacrylamide gel electrophoresis (PAGE) and were subsequently transferred to polyvinylidene fluoride membranes (Millipore). The membranes were blocked in 5% nonfat dry milk dissolved in TBST (150 mM NaCl, 50 mM Tris-HCl, pH 7.5, and 0.05% Tween-20) at room temperature for 1 h and then incubated with primary antibodies against GAPDH (1:1000, Cell Signaling Technology, 5174), TRAF4 (2.5 µg/mL, R&D Systems, 433709), Runx2 (1:1000, Cell Signaling Technology, 8486), OCN (1:1000, Abcam, 13420), Smurf2 (1:1000, Cell Signaling Technology, 12024), Smurf1 (1:1000, Cell Signaling Technology, 2174), Smad1 (1:1000, Cell Signaling Technology, 12430), Flag-Tag (1:1000, Cell Signaling Technology, 14793), Myc-Tag (1:1000, Cell Signaling Technology, 2276), and HA-Tag (1:1000, Cell Signaling Technology, 3724) overnight at 4 °C. After washing 3 times in TBST, the membranes were incubated with the appropriate horse radish peroxidase-conjugated secondary antibodies (1:3000, Beyotime Institute of Biotechnology, A0216, A0208, A0192) for 1 h at room temperature, followed by an additional 3 washes with TBST. The immunoreactive bands on the membranes were detected using chemiluminescent reagents (Millipore) according to the instructions provided by the manufacturer.

### Lentivirus construction and infection

Protocols were reported previously [16]. Briefly, lentiviruses encoding short hairpin RNA (shRNA, GenePharma) targeting TRAF4 (Sh-TRAF4; 5′-GCACTAAGGAGTTCGTCTTTG-3′) and Smurf2 (Sh-Smurf2; 5′-GCAGACCTCTTAGCTGCTTTG-3′) were constructed. The corresponding mutant sequences for TRAF4 (5′-GTACAAAAGAATTTGTTTTCG-3′) and Smurf2 (5′-GAAGGCCACTCAGTTGTTTCG-3′) for RNAi rescue experiments were also constructed. The negative control shRNA sequence was 5′-TTCTCCGAACGTGTCACGTTTC-3′ (NC1). TRAF4 and Smurf2 overexpression lentiviruses and their vector controls (OE-TRAF4, OE-Smurf2, and NC2) were also purchased from GenePharma. Lentiviruses (10^9^ TU/mL) and polybrene (5 μg/mL, Sigma) were added to the medium and incubated with MSCs for 24 h at a multiplicity of infection (MOI) of 30. For simultaneous transfection of Sh-TRAF4/Sh-Smurf2 or OE-TRAF4/OE-Smurf2, the lentiviruses were added at an MOI of 30, and the total amounts of transfected lentiviruses in the other group were equalized by adding the NC lentiviruses.

### CCK-8 assay

MSCs were seeded in 96-well plates containing OM. The proliferation rate of the MSCs was detected using a Cell Counting Kit-8 (Dojindo) according to the protocol. Medium without cells were used as a negative control.

### Quantitative real-time PCR

Quantitative real-time PCR (qRT-PCR) was performed as previously described [16]. Briefly, total RNA was isolated from MSCs using TRIzol (Invitrogen) and was transcribed into cDNA using the PrimeScriptTM RT reagent kit (Takara). qRT-PCR was then performed on a LightCycler^®^480 PCR System (Roche) using SYBR^®^ Premix Ex TaqTM (Takara). The relative expression levels of Smurf2 were determined using the 2^−ΔΔCt^ method. Specific primers for Smurf2 and GAPDH are listed in Supplemental Table 1.

### Plasmid construction and transfection

Expression plasmid constructs, including full-length pcDNA3.1(+)-Flag-Smurf2 (*Homo sapiens* (human)), full-length pcDNA3.1(+)-Myc-TRAF4 (*Homo sapiens*), full-length pcDNA3.1(+)-HA-Ubiquitin (ubiquitin B, UBB, *Homo sapiens*), full-length pcDNA3.1(+)-HA-K48-Ubiquitin (ubiquitin B, UBB, *Homo sapiens*), pcDNA3.1(+)-HA-K63-Ubiquitin (ubiquitin B, UBB, *Homo sapiens*), pcDNA3.1(+)-Myc-TRAF4-RING domain deletion (pcDNA3.1(+)-Myc-TRAF4-RD, 18–58 amino acid deletion, *Homo sapiens*) and pcDNA3.1(+)-Myc-TRAF4-TRAF domain deletion (pcDNA3.1(+)-Myc-TRAF4-TD, amino acids 307–462 deletion, *Homo sapiens*), pcDNA3.1(+)-Flag-Smurf2 catalytic inactive mutant (cysteine 716 to alanine, *Homo sapiens*), and pcDNA3.1(+)-Flag-Smurf2 K119R (lysine 119 to alanine, *Homo sapiens*) were all constructed by and purchased from Obio Technology (Shanghai) Corp., Ltd. A Lipofectamine 3000 Transfection Kit (Invitrogen, L3000-015) was used for transfection. Transfection was performed according to the manufacturer’s instructions with minor modifications. Briefly, 293T cells were seeded in 6-well plates at a density of 3 × 10^5^ cells/well. The use of each plasmid was 2.5 µg/well with 5 µl Lipo3000 and 5 µl P3000 according to the manufacturer’s instructions. The total amount of transfected plasmids in each well was equalized by adding empty pcDNA3.1(+)-vector.

### Coimmunoprecipitation and LC–MS/MS

MSCs or 293T cells were quickly harvested and homogenized on ice in modified RIPA buffer containing 50 mM Tris-HCl (pH 7.5), 150 mM NaCl, 0.1% (vol/vol) Triton X-100, 0.5% (wt/vol) sodium deoxycholate, 0.1% (wt/vol) SDS, 1 mM EDTA, 50 mM N-ethylmaleimide, 1 mM NaF, 1 mM Na_3_VO_4_, 1 mM PMSF, and 1 μg/mL each of aprotinin, leupeptin, and pepstatin. The cell extracts (approximately 200 μg of total protein) were incubated with an antibody against TRAF4 (2 µg, Santa Cruz, sc-390232), Smurf2 (4 µl, Cell Signaling Technology, 12024), Flag-Tag (4 µl, Cell Signaling Technology, 14793), Myc-Tag (1:1000, Cell Signaling Technology, 2276), or their IgG control (Cell Signaling Technology, 3452 or 37988) at 4 °C overnight. Then, protein-G agarose beads (40 L, Beyotime Biotechnology) were added, and the mixture was incubated at 4 °C for 3 h. The agarose beads were collected, washed, and resuspended in 60 μL of sample buffer containing 50 mM Tris-HCl, pH 7.6, 2% (wt/vol) SDS, 10% (vol/vol) glycerol, 10 mM DTT, and 0.2% bromophenol blue. Afterward, the samples were boiled for 10 min. SDS-PAGE was used to separate the samples, and a Coomassie blue staining kit (Beyotime Institute of Biotechnology) was subsequently used. Differential bands were collected for further liquid chromatography with tandem mass spectrometry (LC–MS/MS) to analyze the interaction proteins of TRAF4 in MSCs and the ubiquitination sites of Smurf2. The entire LC–MS/MS procedure was performed by Applied Protein Technology (ATP) company. Western blot protocols were reported above. The special secondary antibody (1:1000, Abcam, ab131366), which only recognizes native (nonreduced) antibodies to highly minimize the detection of heavy and light chains, was used to test the IP samples to avoid the influence of the IP antibodies in the IP samples.

### Immunofluorescence staining

MSCs were seeded on sterile glass coverslips. When cells reached appropriate confluence, the growth medium was aspirated. Cells were washed 3 times with PBS before fixing in 4% paraformaldehyde for 30 min. To permeabilize the cells, 0.1% Triton X-100 was added for 15 min at room temperature, and 5% normal goat serum was used to block cells for 30 min. A primary antibody against TRAF4 (1:50, Abcam, ab88612) or Smurf2 (1:200, Abcam, ab94483) was added, and the cells were incubated overnight at 4 °C. After the cells were washed 3 times with PBS, anti-rabbit IgG (1:500, Cell Signaling Technology, 4413) and anti-mouse IgG (1:500, Cell Signaling Technology, 4408) were added, and the cells were incubated for another 1 h at room temperature. 4′,6-diamidino-2-phenylindole (DAPI) was used to counterstain the nuclei. Thereafter, the coverslips were mounted on glass slides with anti-fade mounting medium (Beyotime, P0126). Thereafter, the samples were viewed under a laser scanning confocal microscope at wavelengths of 488 nm (green, TRAF4), 555 nm (red, Smurf2), and 405 nm (blue, DAPI). Images were obtained using an LSM 5 Exciter confocal imaging system (Carl Zeiss).

### Flag-Smurf2 degradation assay

293T cells were seeded in 6-well plates at a density of 3 × 10^5^ cells/well and transfected with Flag-Smurf2 + empty vector plasmids or Flag-Smurf2 + Myc-TRAF4 plasmids for 36 h. Protein lysates were prepared at the indicated time points after the addition of cycloheximide (CHX) (10 μM). Equal amounts of protein were separated by SDS-PAGE. Levels of Flag-Smurf2 were determined by immunoblotting and quantified at the indicated time points.

### Autophagy–lysosome and ubiquitin–proteasome pathway blocking

293T cells were seeded in 6-well plates at a density of 3 × 10^5^ cells/well and transfected with Flag-Smurf2 and Myc-TRAF4 plasmids for 36 h. Cells were treated with chloroquine (CQ, 50 µM, Cell Signaling Technology, 14774) or MG132 (10 µM, Selleck, S2619) for another 12 h to block the autophagy–lysosome or ubiquitin–proteasome pathway. Protein lysates were harvested after that, and the protein level of Flag-Smurf2 was evaluated by western blot.

### Bone formation of MSCs in vivo

MSCs at the fourth passage infected with lentiviruses (NC1, Sh-TRAF4, NC2, and OE-TRAF4) were cultured in OM for 7 days prior to the in vivo study. After being trypsinized and resuspended directly in DMEM, MSCs (5 × 10^5^) were loaded onto 40 mg hydroxyapatite/tricalcium phosphate (HA/TCP; Zimmer) and then implanted into the dorsal subcutaneous space on the two symmetrical sites of 8-week-old BALB/c-nu/nu female mice (Laboratory Animal Center of Sun Yat-Sen University). (*n* = 9 per group). Specimens were harvested 8 weeks after implantation, and the animals were euthanized by CO_2_ asphyxiation. The bone constructs were fixed in 4% paraformaldehyde and then decalcified for 10 days in 10% EDTA (pH 7.4). After decalcification, the specimens were dehydrated and subsequently embedded in paraffin. Sections (5-mm thickness) were stained with hematoxylin and eosin (H&E) and Masson’s trichrome stain.

### Establishment of ovariectomized rats

Two-month-old female Sprague–Dawley (SD) rats, weighing 200–225 g, were purchased from the Laboratory Animal Center of Sun Yat-Sen University (*n* = 9 per group). Rats were maintained in a pathogen-free facility on a 12-h light/dark cycle with water and food provided ad libitum. Rats were sham-operated (sham) or ovariectomized (OVX). The OVX rats presented osteoporosis as described previously [23]. Rats were allowed 3 months to recover from the ovariectomy surgery and then sacrificed for the related assays [24].

To evaluate the mass and microarchitecture of the bone between different groups, micro-CT was performed using an Inveon MM system (Siemens). Images were acquired at an effective pixel size of 8.82 lm, a voltage of 80 kV, a current of 500 lA and an exposure time of 1500 ms in each of the 360 rotational steps. The parameters of bone volume/total volume (BV/TV) and trabecular number (Tb.N) in the trabecular region (1–2 mm distal to the proximal epiphysis) were calculated using an Inveon Research Workplace (Siemens) according to the guidelines set by the American Society for Bone and Mineral Research. In addition, sections (5-mm thickness) of femur specimens from the different groups were stained with H&E to evaluate bone loss in OVX rats.

### Femur head section biopsies

We recruited six female patients with fractures of the neck of the femur who had been diagnosed with postmenopausal osteoporosis and six female patients with developmental dysplasia of the hip as a control group. All of the patients needed treatment for total hip replacement arthroplasty. The femur head specimen was obtained during the surgery. The characteristics of the study subjects are presented in Supplemental Table 2.

### Immunofluorescence staining of femur bone sections

Femur bone sections from rats and humans were fixed in 4% paraformaldehyde at 37 °C under constant agitation for 3 days. Bones were then decalcified in 10% EDTA (pH 7.4) at 37 °C for 10 days (fresh 10% EDTA solution was exchanged every 48 h). Bones were washed in PBS for 2 h, soaked in 30% sucrose in PBS at 4 °C under constant agitation overnight and finally embedded in paraffin. Sections (5-mm thickness) were stained for immunofluorescence as described previously with minor modifications. Antibodies against TRAF4 (1:100, Thermo Fisher, PA5-77113), Smurf2 (1:100, Novus, NBP2-57554), Smad1 (1:100, Abcam, ab131550), Runx2 (1:100, Abcam, ab23981), and OCN (1:100, Abcam, ab13420) were incubated with the samples overnight at 4 °C. The secondary antibodies used were anti-rabbit IgG (1:500, Cell Signaling Technology, 4413) and anti-mouse IgG (1:500, Cell Signaling Technology, 4408). DAPI was used to counterstain the nuclei. The samples were viewed under a laser scanning confocal microscope at wavelengths of 488 nm (green, OCN), 555 nm (red, TRAF4, Smurf2, Smad1, and Runx2) and 405 nm (blue, DAPI). Images were obtained using an LSM 5 Exciter confocal imaging system (Carl Zeiss).

### Statistical analysis

All results were determined based on three separate experiments, and each separate experiment in MSCs included MSCs from three different donors. All data are expressed as the mean ± standard deviation. Statistical analysis was performed using SPSS 18.0 software (SPSS, Chicago, IL, USA). Student’s *t* test and one-way analysis of variance followed by the Bonferroni test and the Pearson correlation test were performed for statistical analyses. *p* < 0.05 was considered statistically significant.

## Results

### TRAF4 is upregulated during the osteogenic differentiation of MSCs

**Fig. 1:**
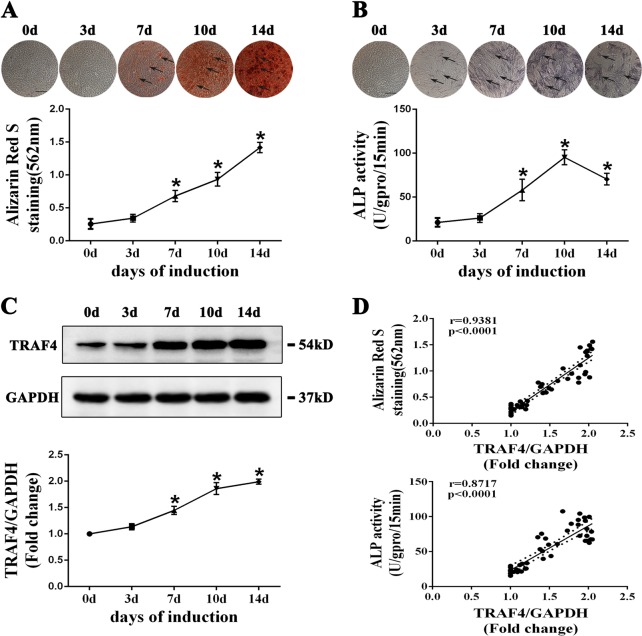
TRAF4 is upregulated during the osteogenic differentiation of MSCs. MSCs were cultured in osteogenic medium for up to 14 days. **a** ARS staining (scale bar = 250 µm) showed a gradual increase in calcium nodule formation from days 0 to 14 (black arrows). **b** ALP staining (scale bar = 250 µm) peaked at 10 days and decreased thereafter (black arrows). **c** The protein level of TRAF4 was upregulated after induction toward the osteogenic lineage and remained at a high level during osteogenesis. **d** TRAF4 protein expression exhibited a strong positive correlation with ARS quantification and ALP activity during the osteogenic differentiation of MSCs. All data are presented as the means ± SD. *p < 0.05 (*n* = 3 independent experiments with three different MSC lines)

We first cultured MSCs in OM for up to 14 days, and ARS and ALP assays were used to determine their osteogenic differentiation potential. Consistent with our previous results, ARS staining revealed a gradual increase in calcium nodule formation from days 0 to 14 (Fig. 1a, black arrows), while ALP staining peaked at 10 days and decreased thereafter (Fig. 1b, black arrows) [16]. The results of western blotting demonstrated that TRAF4 expression was upregulated after induction to the osteogenic lineage and remained at a high level during osteogenesis (Fig. 1c). Moreover, we analyzed the correlation of the expression levels of TRAF4 with ARS and ALP staining and found a strong positive relationship in the osteogenic differentiation of MSCs (Fig. 1d).

### TRAF4 positively regulated the osteogenic differentiation of MSCs both in vitro and in vivo

**Fig. 2:**
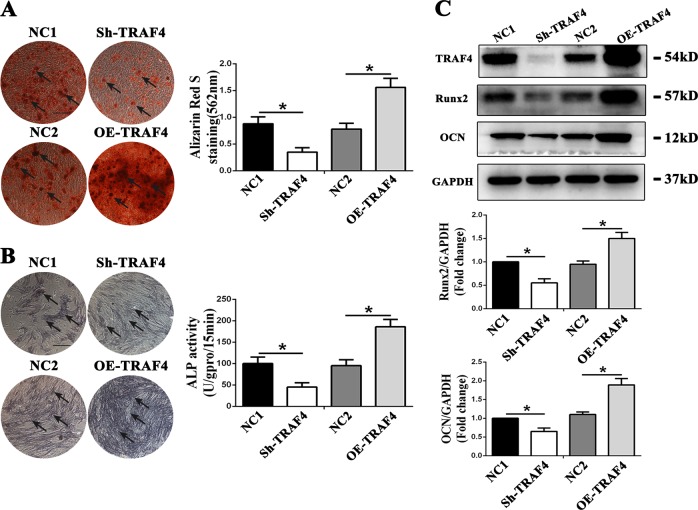
TRAF4 positively regulated the osteogenic differentiation of MSCs in vitro. Lentiviruses were used to diminish and overexpress TRAF4 in MSCs. **a** TRAF4 knockdown by lentiviruses decreased the calcium nodule formation, whereas TRAF overexpression increased calcium nodule formation during osteogenesis of MSCs, as observed through ARS staining (scale bar = 250 µm, black arrows). **b** In ALP assays, the knockdown of TRAF4 led to decreased staining, whereas TRAF4 overexpression led to increased staining during the osteogenesis of MSCs (scale bar = 250 µm, black arrows). **c** Protein levels of the osteogenesis-associated markers Runx2 and OCN were determined by western blot. Diminished TRAF4 in MSCs decreased the expression of Runx2 and OCN during osteogenic induction, while overexpressing TRAF4 had the opposite effect. All data are presented as the means ± SD. *p < 0.05 (*n* = 3 independent experiments with three different MSC lines)

Lentiviruses were used to diminish and overexpress TRAF4 in MSCs (Supplementary Fig. 1A). Neither decrease nor overexpression of TRAF4 in MSCs affected the growth curve of MSCs in the osteogenic medium (Supplementary Fig. 1B). Moreover, to rule out off-target effects, we performed RNAi rescue experiments (Supplementary Fig. 2). After osteogenic induction of MSCs for 14 days, the osteogenic capacity of MSCs in different groups was evaluated. The results of ARS staining demonstrated that knocking down TRAF4 expression decreased calcium nodule formation, whereas the overexpression of TRAF4 increased calcium nodule formation during osteogenesis (Fig. 2a, black arrows). The ALP assay results generally agreed with those obtained with ARS staining, where the knockdown of TRAF4 expression led to decreased and less intense staining, whereas the overexpression of TRAF4 led to denser and darker staining during osteogenesis (Fig. 2b, black arrows). We also examined the expression of osteogenesis-associated markers Runx2 and OCN. The results demonstrated that diminished TRAF4 in MSCs decreased the expression of Runx2 and OCN during osteogenic induction, while overexpressing TRAF4 had the opposite effect (Fig. 2c). In summary, these results indicated that TRAF4 in MSCs promotes the osteogenic differentiation potential of MSCs in vitro.

**Fig. 3:**
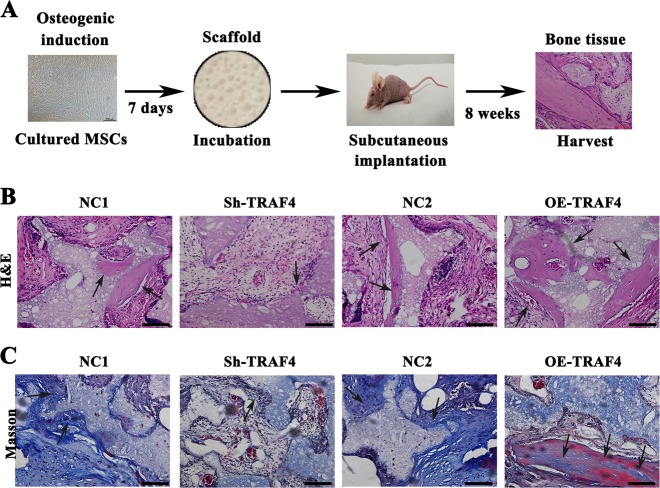
TRAF4 positively regulated the osteogenic differentiation of MSCs in vivo. MSCs were loaded onto scaffolds and implanted in the subcutaneous space of nude mice. **a** Schematic of the experimental setup (*n* = 9 per group). **b** H&E staining (scale bar = 100 µm) revealed that there was less newly formed bone tissue in the Sh-TRAF4 group than in the NC1 group, while there was greatly increased osteoid formation in the OE-TRAF4 group than in the NC2 group (black arrows). **c** Masson’s trichrome staining (scale bar = 100 µm) showed that TRAF4 knockdown significantly reduced the formation of collagen organization (stained blue) compared with that in the NC1 group while TRAF4 overexpression significantly increased bone-like tissue formation compared to that in the NC2 group (black arrows)

MSCs were loaded onto scaffolds and implanted into the subcutaneous space of nude mice (Fig. 3a). The H&E staining results demonstrated that knocking down TRAF4 significantly decreased the formation of new bone tissue, whereas the overexpression of TRAF4 significantly increased new osteoid formation (Fig. 3b, black arrows). The Masson’s trichrome staining results were consistent with those of H&E staining, where the knockdown of TRAF4 led to a decrease in the formation of blue-stained collagen organization while TRAF4 overexpression significantly increased the formation of bone-like tissue (Fig. 3c, black arrows). In summary, TRAF4 positively regulated the osteogenic differentiation of MSCs both in vitro and in vivo.

### TRAF4 diminishes the expression of Smurf2 to modulate osteogenesis of MSCs

**Fig. 4:**
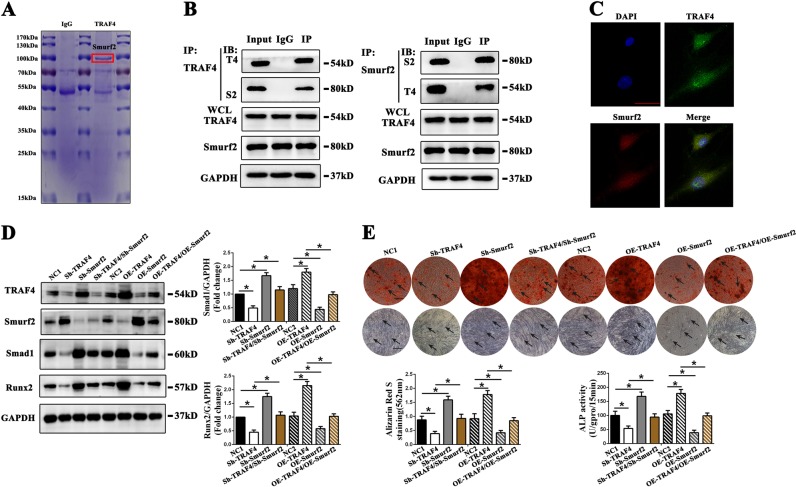
TRAF4 diminished the expression of Smurf2 to modulate the osteogenesis process of MSCs. **a** Coomassie blue staining of the coimmunoprecipitated mixture separated by SDS-PAGE. **b** Coimmunoprecipitated mixtures were separated by SDS-PAGE and evaluated by western blot. Endogenous TRAF4 and Smurf2 in MSCs interacted with each other. **c** Immunofluorescence assays showed the colocalization of TRAF4 and Smurf2 in MSCs (green TRAF4, red Smurf2, blue DAPI). **d** The Smad1 and Runx2 protein levels were decreased in the Sh-TRAF4 group and increased in the Sh-Smurf2 group compared with those in the NC1 group, while compared with Sh-TRAF4, Sh-TRAF4/Sh-Smurf2 rescued the expression of Smad1 and Runx2. The Smad1 and Run2 protein levels increased in the OE-TRAF4 group and decreased in the OE-Smurf2 group compared with the NC2 group, while the expression levels of Smad1 and Runx2 in the OE-TRAF4/OE-Smurf2 group was decreased compared with those observed in the OE-TRAF4 group. **e** The simultaneous knockdown of TRAF4 and Smurf2 increased the ARS and ALP staining compared with that in the Sh-TRAF4 group (scale bar = 250 µm, black arrows), while the simultaneous overexpression of TRAF4 and Smurf2 decreased the ARS and ALP staining to the level observed in the NC2 group compared with OE-TRAF4 group (scale bar = 250 µm, black arrows). Data in (**d**) and (**e**) are presented as the means ± SD. *p < 0.05 (*n* = 3 independent experiments with three different MSC lines)

Accumulating studies have demonstrated that TRAF4 interacts with several molecules to function in certain circumstances [25, 26]. Thus, we speculated that TRAF4 may interact with some classical osteogenesis-related molecules to modulate the osteogenic differentiation of MSCs. We first used coimmunoprecipitation (Co-IP) and LC–MS/MS experiments to identify the proteins that interact with TRAF4 during the osteogenesis of MSCs. We identified 277 proteins that may interact with TRAF4 during the osteogenesis of MSCs (Supplementary Document 1). Furthermore, we noticed that Smurf2 was involved in the Co-IP complex (Fig. 4a). Smurf2 is a classical negative regulator of the osteogenic differentiation process of MSCs, and previous studies have confirmed that TRAF4 can interact with Smurf2 in several contexts [27, 28]. We conducted reciprocal Co-IP/western blot assays to confirm again that endogenous TRAF4 and Smurf2 interact with each other in MSCs (Fig. 4b) and that exogenous Myc-TRAF4 and Flag-Smurf2 interact with each other in 293T cells (Supplementary Fig. 3A). Furthermore, immunofluorescence assay revealed the colocalization of TRAF4 and Smurf2 in MSCs (Fig. 4c).

We further evaluated the level of Smurf2 after modulation the expression of TRAF4. The results demonstrated that knocking down TRAF4 increased the expression of Smurf2, while overexpressing TRAF4 decreased the expression of Smurf2 in MSCs during the osteogenesis process at the protein level but not at the mRNA level (Supplementary Fig. 3B). Moreover, we assessed the mRNA and protein levels of TRAF4, Smurf2, and the related Smurf1 during the osteogenic differentiation of MSCs. The results demonstrated that the mRNA levels of both TRAF4 and Smurf2 remained stable during osteogenic differentiation (Supplementary Fig. 3C), whereas the level of TRAF4 protein was elevated and that of Smurf2 was decreased during the osteogenic differentiation process (Fig. 1c and Supplementary Fig. 3D). In addition, both mRNA and protein levels of the related Smurf1 remained unchanged during the osteogenic differentiation process (Supplementary Fig. 3C and D). Previous studies have confirmed that Smurf2 negatively regulates the osteogenesis potential of MSCs primarily through the degradation of Smad1 and Runx2. Knocking down Smurf2 by lentivirus increased Smad1 and Runx2 expression, whereas overexpression of Smurf2 decreased Smad1 and Runx2 in MSCs during osteogenesis (Supplementary Fig. 3E). Further studies demonstrated that the expression levels of Smad1 and Runx2 were decreased in the Sh-TRAF4 group but increased in the Sh-Smurf2 group, whereas simultaneous knockdown TRAF4 and Smurf2 rescued the expression of Smad1 and Runx2 compared with that in the Sh-TRAF4 group (Fig. 4d). Overexpression of TRAF4 increased Smad1 and Runx2, whereas Smurf2 overexpression decreased Smad 1 and Runx2 and simultaneous overexpression of TRAF4 and Smurf2 decreased Smad1 and Runx2 to those observed in the NC2 group compared with the OE-TRAF4 group (Fig. 4d).

Further studies demonstrated that the simultaneous knockdown of TRAF4 and Smurf2 rescued the ARS and ALP assays compared with that in the Sh-TRAF4 group, whereas simultaneous overexpression of TRAF4 and Smurf2 decreased ARS and ALP assays to the level observed in the NC2 group compared with OE-TRAF4 group (Fig. 4e, black arrows). In summary, these data indicated that TRAF4 decreased the expression of Smurf2 during the osteogenesis process to regulate the osteogenesis potential of MSCs.

### TRAF4 induced the degradation of Smurf2 in a ubiquitin/proteasome-dependent manner, TRAF4 binds with Smurf2 through the TRAF domain, and its degradation function on Smurf2 relies on both the RING and TRAF domains

**Fig. 5:**
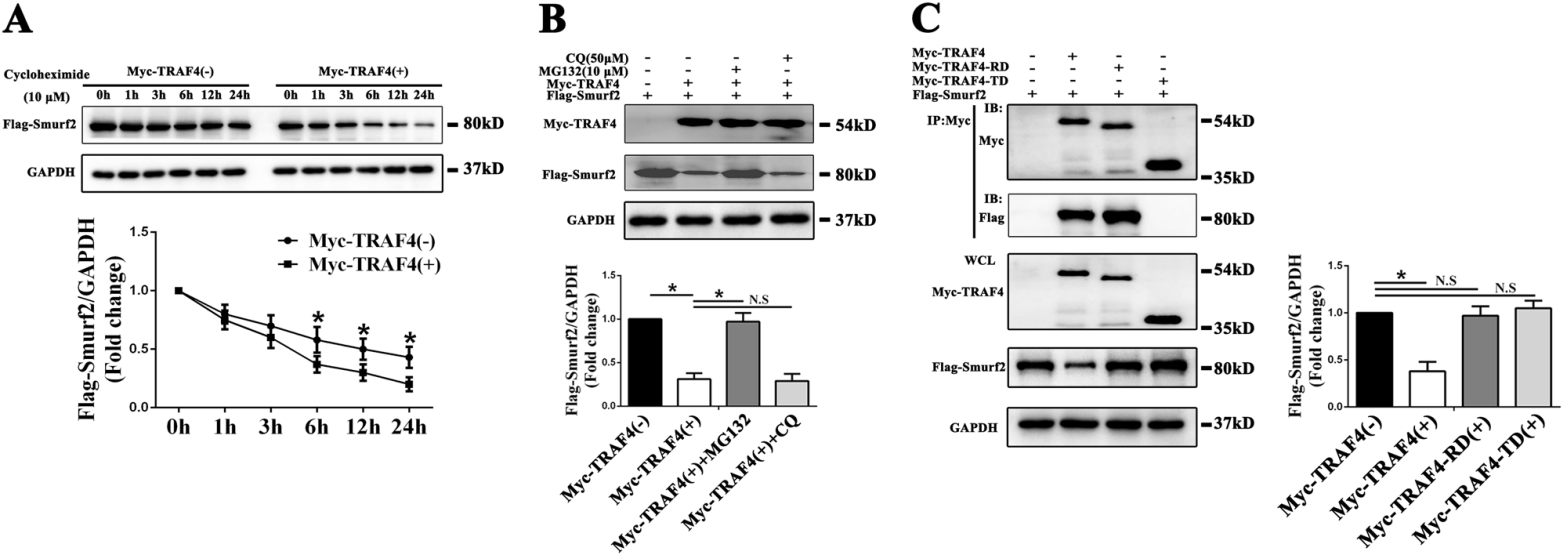
TRAF4 induced the degradation of Smurf2 in a ubiquitin/proteasome-dependent manner, TRAF4 binds with Smurf2 through the TRAF domain, and its degradation function on Smurf2 relies on both the RING and TRAF domains. **a** Flag-Smurf2 was transfected into 293T cells with Myc-TRAF4 or empty vectors. After transfection of plasmids for 36 h, protein lysates were prepared at the indicated time points after treatment with 10 μM cycloheximide, and the Flag-Smurf2 protein levels were evaluated by western blot. The results demonstrated that the Flag-Smurf2 degradation rate was higher when coexpressed with Myc-TRAF4. **b** Flag-Smurf2 and Myc-TRAF4 were coexpressed in 293T cells for 36 h, after which 293T cells were treated with chloroquine (CQ, 50 µM) or MG132 (10 µM) for another 12 h. Western blotting demonstrated that MG132 can reverse the degradation of Flag-Smurf2-mediated by Myc-TRAF4, while CQ had little effect. **c** After coexpressing the TRAF4-WT or TRAF4 mutants with Flag-Smurf2 into 293T cells, Myc-TRAF4 was immunoprecipitated with anti-Myc, and Flag-Smurf2 was detected in the IP complex by western blotting. The results demonstrated that the TRAF domain mediated the interaction between TRAF4 and Smurf2, and both the RING domain deletion mutant and the TRAF domain deletion mutant caused TRAF4 to lose the ability to degrade Flag-Smurf2. All data are presented as the means ± SD. *p < 0.05 (*n* = 3 independent experiments)

As mentioned above, TRAF4 diminished the expression of Smurf2 at the protein level but not at the mRNA level. We speculated that TRAF4 might affect the degradation of Smurf2 protein. We then evaluated the degradation kinetics of Flag-Smurf2 protein and the results demonstrated that the Flag-Smurf2 degradation rate was higher when coexpressed with Myc-TRAF4 (Fig. 5a). Proteins were primarily degraded through the autophagy–lysosome pathway or the ubiquitin–proteasome pathway. Thus, we added chloroquine (CQ, 50 µM) or MG132 (10 µM) to block the autophagy–lysosome pathway [29] or the ubiquitin–proteasome pathway [30], respectively. The results demonstrated that MG132 can reverse the degradation of Flag-Smurf2-mediated by Myc-TRAF4, while CQ had little effect (Fig. 5b). This result indicated that TRAF4 degraded Smurf2 through the ubiquitin–proteasome pathway instead of the autophagy–lysosome pathway.

We constructed Myc-TRAF4-RING domain deletion and Myc-TRAF4-TRAF domain deletion mutants and overexpressed with Flag-Smurf2 in 293T cells. Myc-TRAF4 was immunoprecipitated with anti-Myc, and Flag-Smurf2 was detected in the IP complex. The results showed that the TRAF domain mediated the interaction between TRAF4 and Smurf2 (Fig. 5c). We also showed that the RING domain deletion mutant or the TRAF domain deletion mutant lost the ability to degrade Flag-Smurf2 (Fig. 5c). Collectively, our data demonstrated that TRAF4 binds with Smurf2 through the TRAF domain and that its degradation function of Smurf2 relies on both RING and TRAF domains.

### TRAF4 acts as an E3 ubiquitin ligase to ubiquitinate the K119 site of Smurf2 through the K48 ubiquitin chain and degrade Smurf2

**Fig. 6:**
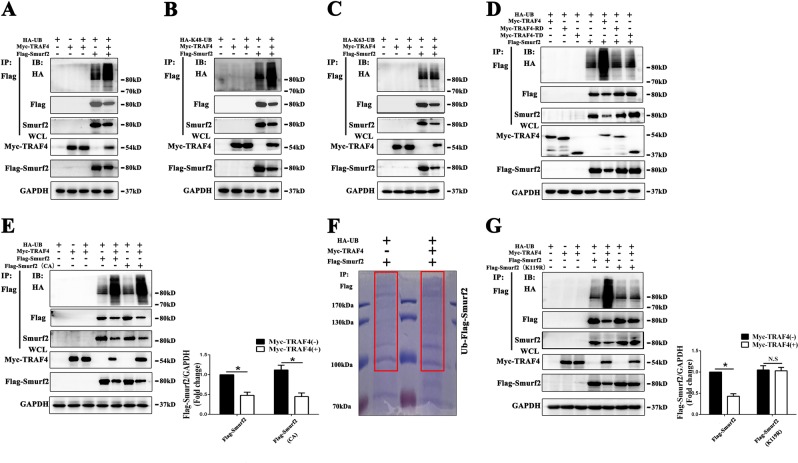
TRAF4 acts as an E3 ubiquitin ligase to ubiquitinate the K119 site of Smurf2 through the K48 ubiquitin chain and degrade Smurf2. After coexpression of HA-Ubiquitin, Myc-TRAF4 and Flag-Smurf2 into 293T cells for 36 h, cell lysates were harvested, and Flag-Smurf2 was immunoprecipitated with anti-Flag. **a** Western blotting demonstrated that Myc-TRAF4 significantly induced the ubiquitination of Flag-Smurf2. **b**, **c** Myc-TRAF4 significantly induced K48-linked ubiquitination of Flag-Smurf2 rather than K63-linked ubiquitination. **d** Deletion of either the RING domain or the TRAF domain from TRAF4 caused TRAF4 to lose the ability to induce the ubiquitination of Smurf2. **e** A catalytically inactive mutant of Smurf2 (cysteine 716 to alanine) did not affect the TRAF4-dependent ubiquitination and degradation of Smurf2. **f** Coomassie blue staining of immunoprecipitated Flag-Smurf2 coexpressed with Myc-TRAF4 or empty vector separated by SDS-PAGE. **g** Mutation of the 119 lysine residues to arginine (K119R) significantly reduced TRAF4-mediated ubiquitination of Smurf2, and TRAF4 lost the ability to degrade the expression of Smurf2 (K119R). The results shown in (**e**) and (**g**) are presented as the means ± SD. *p < 0.05 (*n* = 3 independent experiments)

We coexpressed HA-Ubiquitin, Myc-TRAF4 and Flag-Smurf2 into 293T cells and observed that Myc-TRAF4 significantly induced the ubiquitination of Flag-Smurf2 (Fig. 6a). Furthermore, TRAF4-mediated K48-linked ubiquitination of Smurf2 instead of K63-linked ubiquitination (Fig. 6b, c). Deletion of either RING domain or TRAF domain from TRAF4 caused TRAF4 to lose the ability to induce ubiquitination of Smurf2 (Fig. 6d). As Smurf2 can autoubiquitinate and degrade itself [31], to exclude the possibility that TRAF4-dependent ubiquitination and degradation of Smurf2 was due to Smurf2 autoubiquitination, we constructed a plasmid with catalytically inactive mutant Smurf2 (cysteine 716 to alanine) [31]. The results demonstrated that the catalytically inactive Smurf2 mutant did not affect TRAF4-dependent ubiquitination and degradation of Smurf2 (Fig. 6e), indicating that TRAF4-dependent ubiquitination and degradation of Smurf2 is not due to Smurf2 autoubiquitination. To further explore the precise lysine site in Smurf2 for TRAF4-mediated ubiquitination, we conducted IP and LC–MS/MS experiments. Samples stained with Coomassie blue are shown in Fig. 6f. The results indicated that Myc-TRAF4 coexpression significantly induced ubiquitination of K119 site of Smurf2 compared with that observed in cells without Myc-TRAF4 expression (Supplementary Document 2). We then used site-directed mutagenesis to generate lysine to arginine mutants of Smurf2 (K119R). The mutation significantly reduced TRAF4-mediated ubiquitination of Smurf2 (Fig. 6g), and TRAF4 lost ability to decrease the expression of Smurf2 (K119R) (Fig. 6g). In summary, these data indicated that TRAF4 may act as an E3 ubiquitin ligase to ubiquitinate K119 site of Smurf2 through K48 ubiquitin chain and degrade Smurf2.

### TRAF4 is decreased in bone sections of osteoporotic rats and patients

**Fig. 7:**
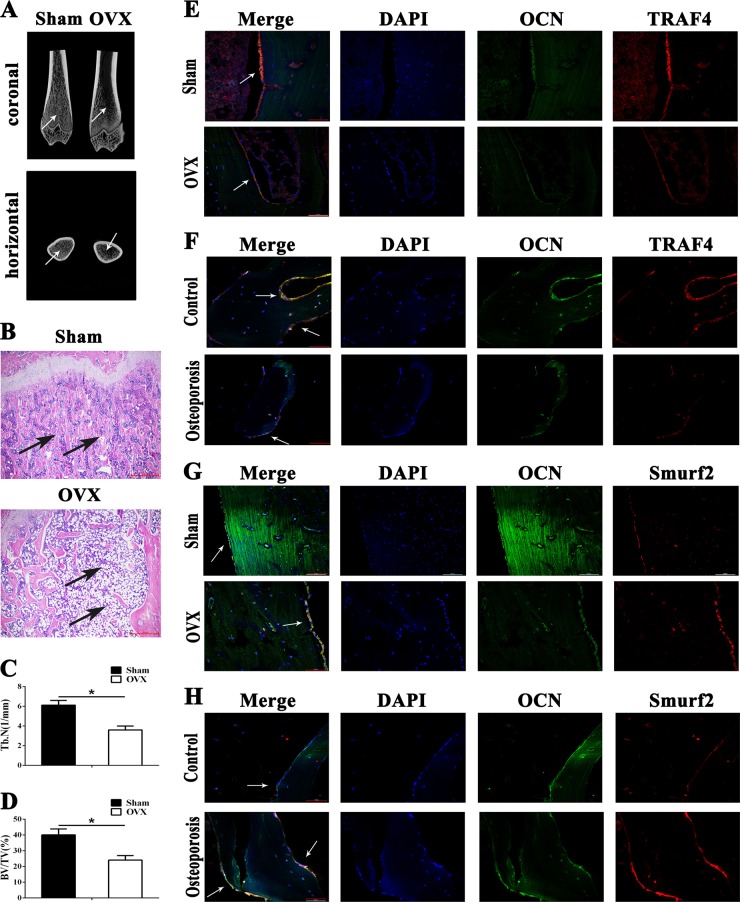
TRAF4 is decreased in bone sections of osteoporotic rats and patients. To evaluate the possible functional significance of TRAF4 in bone remodeling in vivo, we produced OVX rats as an animal model of postmenopausal osteoporosis. **a** Representative micro-CT image showing trabecular bone loss in OVX rats compared with the sham-operated rats (white arrows). **b** Representative H&E staining (scale bar = 100 µm) of trabecular bone loss in OVX rats compared with the sham-operated rats (black arrows). **c** Trabecular number was reduced in OVX rats. **d** Bone volume was reduced in OVX rats. **e** Immunofluorescence staining (scale bar = 50 µm) demonstrated that TRAF4 was weaker in the OCN^+^ osteoblast lineage in OVX rats than in sham-operated rats (white arrows). **f** Immunofluorescence staining (scale bar = 50 µm) demonstrated that the expression of TRAF4 was weaker in the OCN^+^ osteoblast lineage in osteoporotic patients than in control patients (white arrows). **g** Immunofluorescence staining (scale bar = 50 µm) demonstrated that Smurf2 expression was increased in the OCN^+^ osteoblast lineage in OVX rats compared to the sham-operated rats (white arrows). **h** Immunofluorescence staining (scale bar = 50 µm) demonstrated that Smurf2 expression was increased in the OCN^+^ osteoblast lineage in osteoporotic patients compared to the control patients (white arrows)

Osteoporosis is one of the most representative bone metabolism disorders [32]. To evaluate the possible functional significance of TRAF4 in bone metabolism in vivo, we produced OVX rats as an animal model of postmenopausal osteoporosis [33]. The results of both micro-CT (Fig. 7a, white arrows) and H&E staining (Fig. 7b, black arrows) showed that the level of trabecular bone was significantly reduced in OVX rats compared with sham-operated rats (Fig. 7a–d). Next, we examined whether the expression of TRAF4 was impaired in osteoblasts during osteoporosis in bone sections using immunofluorescence staining. TRAF4 expression was observed to be weaker in the OCN^+^ osteoblast lineage in OVX rats than in sham-operated rats (Fig. 7e, white arrows). Furthermore, we collected femur specimens from osteoporotic patients and age-matched controls. The results demonstrated that the expression of TRAF4 was also lower in the OCN^+^ osteoblast lineage in osteoporotic patients than in the control patients (Fig. 7f, white arrows). Moreover, we further explored the expression of Smurf2, Smad1, and Runx2 in the bone samples, and the results demonstrated that the expression of Smurf2 was stronger (Fig. 7g, h, white arrows), while that of Smad1 and Runx2 was weaker (Supplementary Fig. 4, white arrows) in the OCN^+^ osteoblast lineage in OVX rats and osteoporotic patients than in the control samples. Taken together, these results indicated that the expression of TRAF4–Smurf2 axis in the osteoblast lineage was abnormal in osteoporosis.

## Discussion

**Fig. 8:**
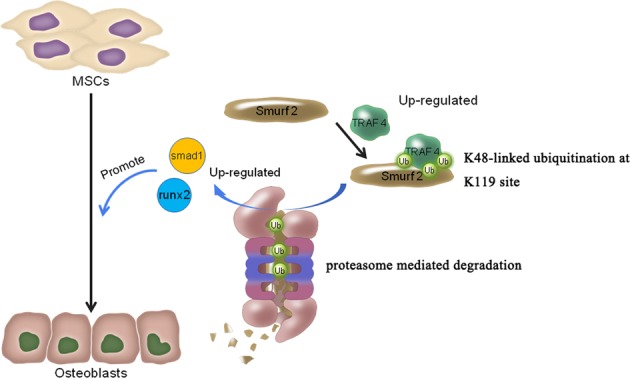
A schematic presentation of the functions of TRAF4 on osteoblast differentiation. TRAF4 positively regulated the osteogenic process of MSCs by acting as an E3 ubiquitin ligase to mediate the K48-linked ubiquitination of Smurf2 at the K119 site and cause degradation

In this study, we evaluated TRAF4 during the process of osteogenic differentiation of MSCs and observed that TRAF4 positively regulated the osteogenic process both in vitro and in vivo. We further demonstrated that TRAF4 regulated the osteogenic process by acting as an E3 ubiquitin ligase to mediate the K48-linked ubiquitination of Smurf2 at K119 site and cause degradation (Fig. 8). In addition, we evaluated the expression of TRAF4 and Smurf2 in femur bone sections of osteoporotic rats and patients and observed that TRAF4–Smurf2 axis was abnormal in the above samples.

TRAF4 is a unique member of the TRAF family [6]. Previous studies have clearly demonstrated that TRAF4 deficiency led to severe skeletal malformations in mice [7]. However, few subsequent studies have focused on the concrete mechanism by which TRAF4 deficiency causes skeletal malformations. Osteoblasts are responsible for building bone [8, 34], and MSCs are the progenitor cells of osteoblasts. Since TRAF deficiency causes severe malformation of skeleton development, we speculated whether TRAF4 is involved in the osteogenic capacity of MSCs. In this study, we evaluated the expression TRAF4 during the osteogenesis of MSCs and observed that manipulating the expression of TRAF4 significantly changes the osteogenic capacity of MSCs both in vitro and in vivo. This result, for the first time, emphasizes the critical roles of TRAF4 not only in the osteogenic differentiation of MSCs but also in skeletal development.

Nevertheless, the question remains regarding how TRAF4 regulates the osteogenic differentiation of MSCs. To further explore the mechanism of TRAF4 in regulating the osteogenic process, we first screened the proteins interacting with TRAF4 in MSCs. We identified Smurf2 as a downstream effector protein in this process. Indeed, several studies have previously demonstrated that TRAF4 interacts with Smurf2 to play an important role in both physiological and pathological conditions. For example, TRAF4 was shown to antagonize Smurf2 to promote metastatic breast cancer cell migration [27]. In our study, we demonstrated that TRAF4 interacts with Smurf2 and that TRAF4 decreases the expression of Smurf2 to promote the osteogenic process. Smurf2 is a member of the Hect domain family of E3 ubiquitin ligases [12], and previous studies have confirmed the important role of Smurf2 in regulating the physiological osteogenic process [12, 14, 15]. However, previous studies have focused only on the downstream effects of Smurf2 during the osteogenic process, as Smurf2 acts as the E3 ubiquitin ligase to degrade Smad1 and Runx2 to negatively regulate the osteogenic process of MSCs [12, 14, 15]. The upstream mechanism that regulates the expression of Smurf2 during the osteogenic process remains unclear. In our study, we demonstrated that TRAF4 is elevated during the osteogenic process of MSCs and that it decreased the expression of Smurf2 to promote the osteogenic process. These findings indicated that TRAF4 may be an important upstream molecule that regulates the expression of Smurf2 during the osteogenic differentiation process of MSCs. Moreover, in our study, we demonstrated that the mRNA level of both TRAF4 and Smurf2 remained stable during osteogenic differentiation, but the level of TRAF4 protein was elevated during this process, whereas that of Smurf2 was decreased. Previous studies have demonstrated that Smurf2 can act as an E3 ubiquitin ligase to promote TRAF4 ubiquitin-mediated degradation [27]. Thus, the results described above lead to an intriguing possibility that the TRAF4–Smurf2 axis may form a positive cycle to promote the osteogenic differentiation of MSCs, as TRAF4 decreased the expression of Smurf2, the Smurf2-mediated TRAF4 degradation decreased and the expression of TRAF4 became further elevated during the osteogenic process to positively regulate the osteogenic capacity of MSCs. Thus, our study supplements existing knowledge regarding the whole regulatory network of the osteogenic process and helps provide a better understanding of the molecular mechanisms underlying MSC osteogenic differentiation.

Zhang et al. [27] previously demonstrated that TRAF4 mediates the degradation of Smurf2 through the ubiquitin–proteasome pathway. Degradation is one of the most common and important types of posttranslational modification for modulating the expression of proteins at a steady level to maintain physiological homeostasis [35, 36], and degradation of proteins can occur through the autophagy–lysosome pathway [37] or the ubiquitin–proteasome pathway [38]. Both types of degradation have been reported to regulate the osteogenic differentiation of MSCs [39–42]. However, a previous study has also demonstrated that TRAF4 can induce the K63-linked ubiquitination of Akt [26], which is known to be involved in the autophagy–lysosome pathway [43, 44]. Thus, it is necessary to explore whether TRAF4 may mediate the degradation of Smurf2 not only through the ubiquitin–proteasome pathway but also through the autophagy–lysosome pathway. In previous study, researchers used only block the ubiquitin–proteasome pathway [27]. However, in our study, we used both MG132 to block the ubiquitin–proteasome pathway and CQ to block the autophagy–lysosome pathway. Our results demonstrated that MG132 could block the ability of TRAF4 to mediate Smurf2 degradation, whereas CQ had little effect. Thus, for the first time, the results of our study excluded the possibility that TRAF4 may mediate the degradation of Smurf2 through the autophagy–lysosome pathway. Moreover, despite Zhang et al. [27] having previously demonstrated that the C2 and WW domains of Smurf2 are of crucial importance for the interaction between TRAF4 and Smurf2, domains responsible for this interaction had not been explored in TRAF4. In our study, we demonstrated that the TRAF domain mediates the interaction between TRAF4 and Smurf2. In addition, we further demonstrated that the RING domain is necessary for the E3 ubiquitin ligase activity of TRAF4 to mediate the ubiquitination and degradation of Smurf2. Li et al. [26] also previously demonstrated that the TRAF domain in TRAF4 is responsible for the interaction between TRAF4 and Akt, while the RING domain is necessary for the K-63 linked ubiquitination of Akt.

Ubiquitination is an important posttranslational modification that regulates protein degradation, trafficking, activity, and protein–protein interactions [45–48]. Previous studies have already demonstrated that the “E3 ubiquitin ligase” characteristics of TRAF4 are of great importance for TRAF4 to function in certain circumstances. Consistent with these findings, Zhang et al. [27] demonstrated that TRAF4 can act as an E3 ubiquitin ligase to modulate the ubiquitination of Smurf2 to promote the migration of metastatic breast cancer. However, they did not explore the type of ubiquitin chain linkage or identify the ubiquitination Lys site in Smurf2. In our study, we demonstrated that TRAF4 mediates K48-linked ubiquitination of Smurf2. Moreover, we further identified Lys 119 in Smurf2 as the primary ubiquitination site mediated by TRAF4. Thus, in this study, we performed an in-depth analysis of the mechanism of Smurf2 ubiquitination mediated by TRAF4. As Smurf2 can autoubiquitinate itself to promote its degradation [31], we constructed a catalytically inactive Smurf2 mutant and indicated that Smurf2 autoubiquitination is not involved in the TRAF4-dependent ubiquitination and degradation of Smurf2. In summary, in this study, we performed an in-depth analysis of the mechanism of TRAF4-mediated Smurf2 ubiquitination and degradation on the basis of the findings of Zhang et al. [12, 24, 27].

MSCs are now considered to be the most promising cell type in tissue engineering technology for bone regeneration and repair. However, the molecular mechanism that governs the osteogenic differentiation of MSCs remains largely unknown, which hampers further development of MSC-based cell therapies for bone defects in the clinic. Thus, our findings indicate that TRAF4 may be a prospective target for improving the efficiency of MSC-based bone repair therapies in the clinic. We also discovered that TRAF4 was abnormally decreased in the femur bone sections of osteoporotic samples. Previous studies have demonstrated that abnormal expression of TRAF4 plays an important role in the pathogenesis of allergic airway inflammation, breast cancer, lung cancer and prostate cancer. Thus, our finding of an abnormal decrease in TRAF4 in osteoporosis indicated that TRAF4 may play an important role in the pathogenesis of bone metabolism disorders, such as osteoporosis. Further studying TRAF4 may provide novel insight for understanding pathogenesis of bone metabolism disorders and reveal new therapeutic targets.

In conclusion, we demonstrated that TRAF4 positively regulated the osteogenic capacity of MSCs. However, there are still some limitations of the present study. To further explore the concrete role of TRAF4 in osteogenesis, transgenic mice that specifically knock in or knockdown TRAF4 in osteoblast lineages are necessary, and we are currently developing these transgenic mice for future studies.

## Supplementary information

Supplementary Figure 1

Supplementary Figure 2

Supplementary Figure 3

Supplementary Figure 4

Supplementary Table 1

Supplementary Table 2

Supplementary Document 1

Supplementary Document 2

Supplementary figure legends
