# Supplementary figures and images for: TRAF4 positively regulates the osteogenic differentiation of mesenchymal stem cells by acting as an E3 ubiquitin ligase to degrade Smurf2
Source: Cell Death Differ. 2019 May 10;26(12):2652–66. doi: 10.1038/s41418-019-0328-3 (PMC7224386; doi:10.1038/s41418-019-0328-3)

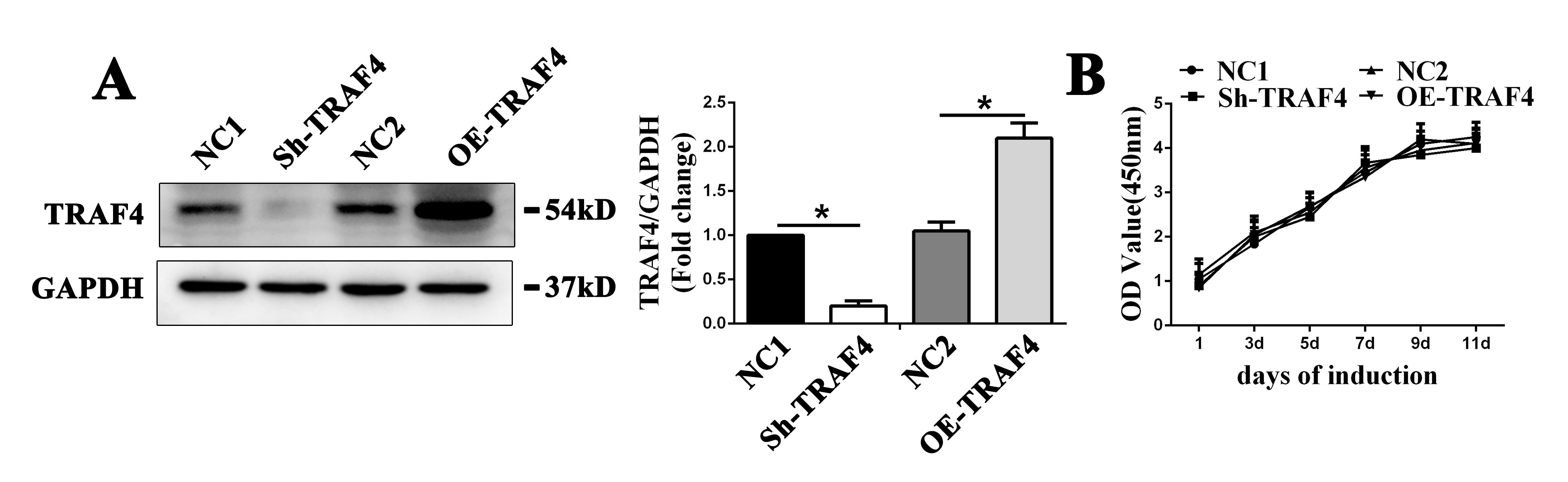

Supplement: Supplementary file 1 — Supplementary Figure 1 [file 41418_2019_328_MOESM1_ESM.jpg]

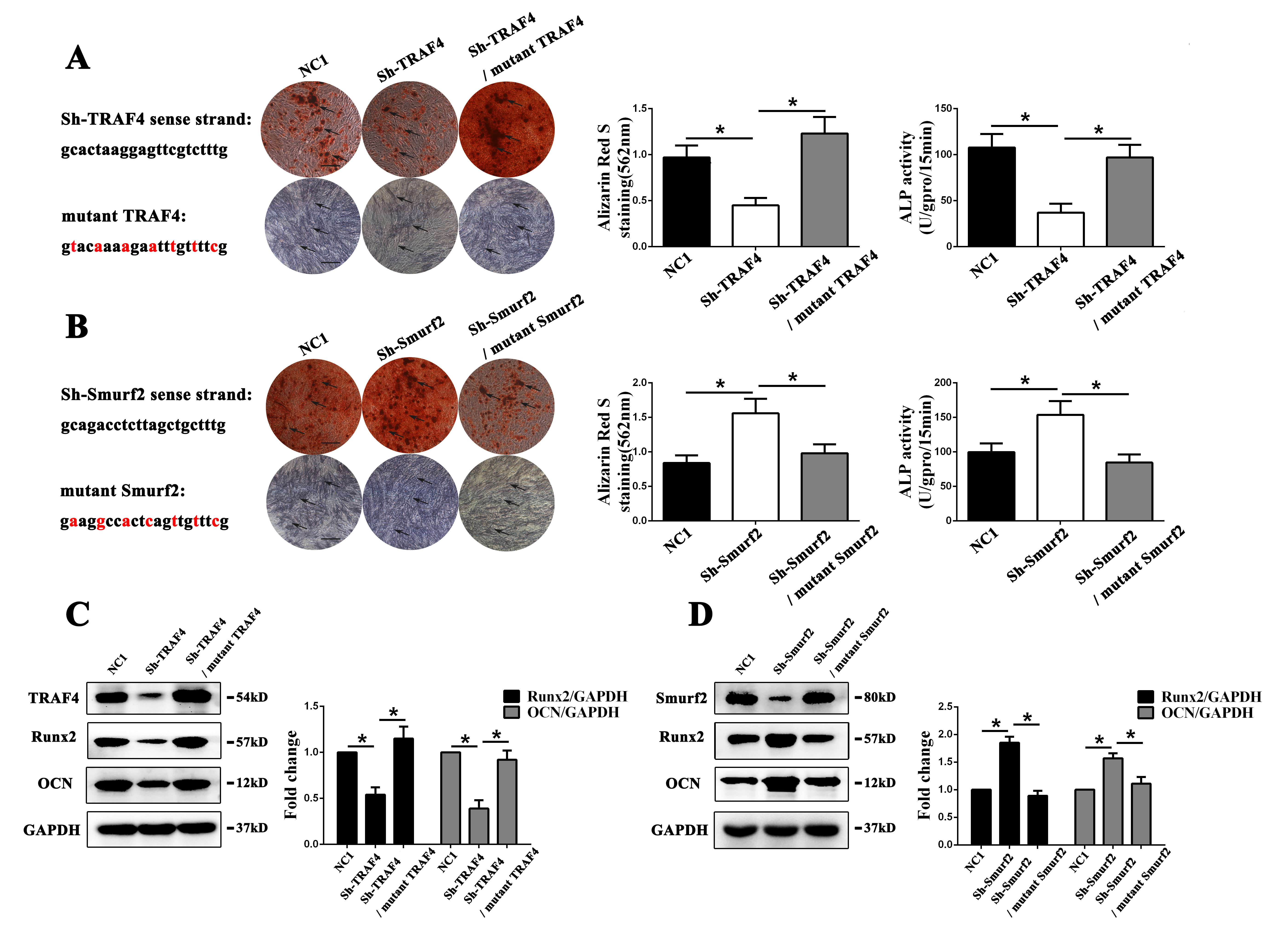

Supplement: Supplementary file 2 — Supplementary Figure 2 [file 41418_2019_328_MOESM2_ESM.jpg]

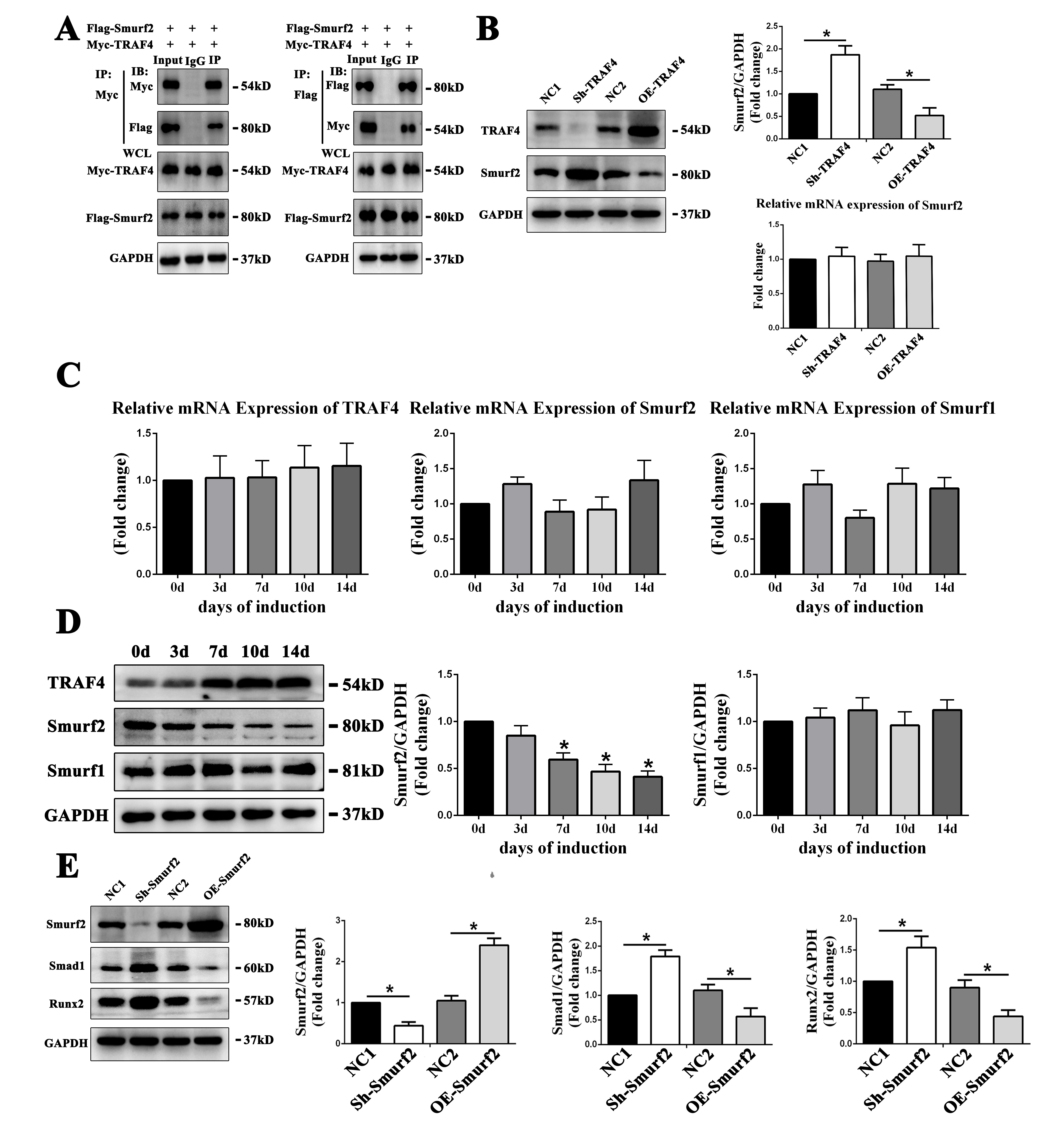

Supplement: Supplementary file 3 — Supplementary Figure 3 [file 41418_2019_328_MOESM3_ESM.jpg]

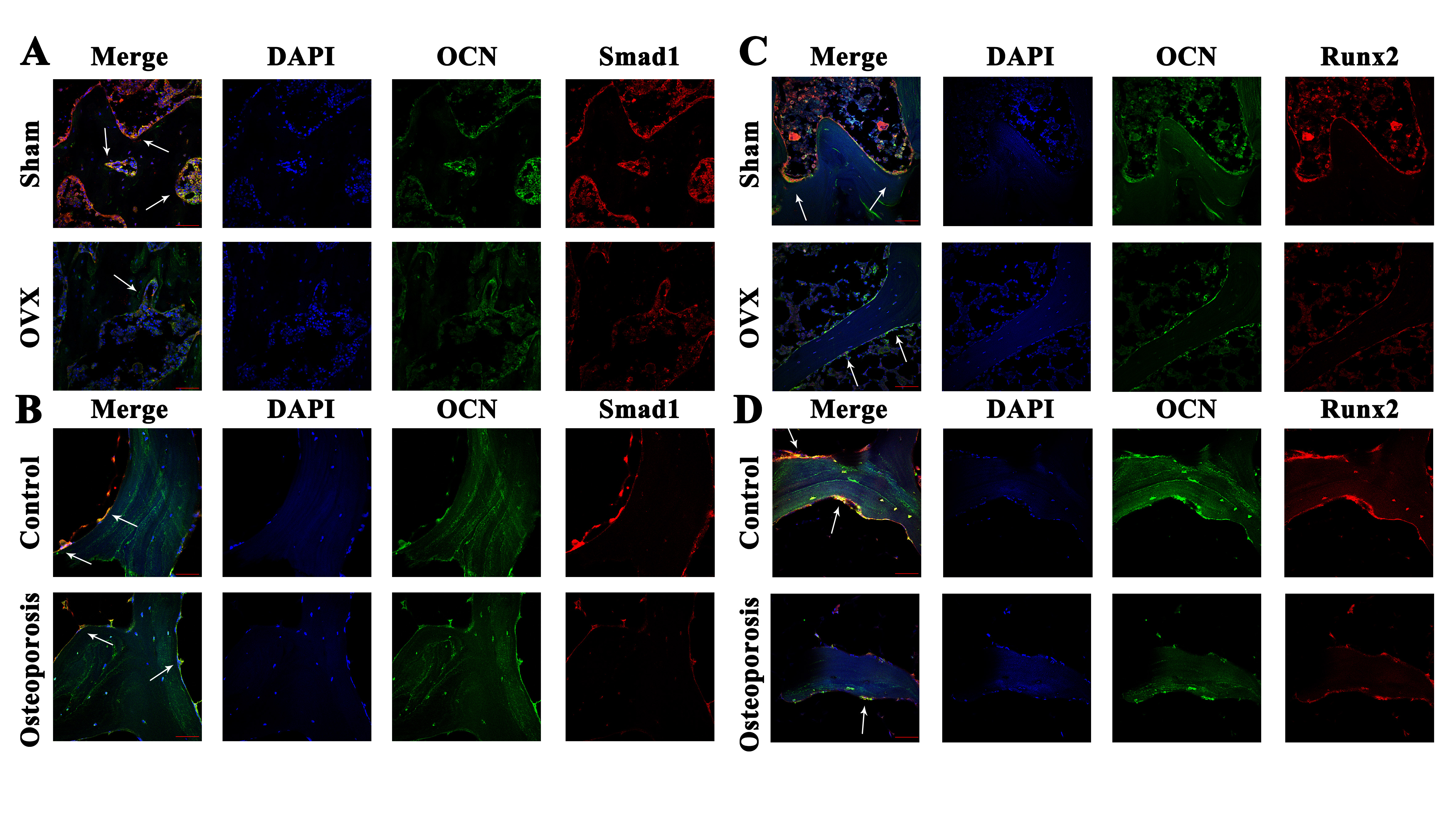

Supplement: Supplementary file 4 — Supplementary Figure 4 [file 41418_2019_328_MOESM4_ESM.jpg]
