# Supplementary material for: TRAF4 positively regulates the osteogenic differentiation of mesenchymal stem cells by acting as an E3 ubiquitin ligase to degrade Smurf2
Source: Cell Death Differ. 2019 May 10;26(12):2652–66. doi: 10.1038/s41418-019-0328-3 (PMC7224386; doi:10.1038/s41418-019-0328-3)
Supplement: Supplementary file 5 — Supplementary Table 1 [file 41418_2019_328_MOESM5_ESM.docx]

**Supplemental Table 1 Primers used for qRT-PCR**

| Gene | NCBI Gene ID | Forward primer  (5’-3’) | Reverse primer  (5’-3’) | Product size (bp) |
| --- | --- | --- | --- | --- |
| Smurf 2 | 64750 | GGCAATGCCATTCTACAGATACT | CAACCGAGAAATCCAGCACCT | 154 |
| Smurf 1 | 57154 | AGATCCGTCTGACAGTGTTATGT | CCCATCCACGACAATCTTTGC | 92 |
| GAPDH | 2597 | GGAGCGAGATCCCTCCAAAAT | GGCTGTTGTCATACTTCTCATGG | 197 |
