# Supplementary material for: TRAF4 positively regulates the osteogenic differentiation of mesenchymal stem cells by acting as an E3 ubiquitin ligase to degrade Smurf2
Source: Cell Death Differ. 2019 May 10;26(12):2652–66. doi: 10.1038/s41418-019-0328-3 (PMC7224386; doi:10.1038/s41418-019-0328-3)
Supplement: Supplementary file 6 — Supplementary Table 2 [file 41418_2019_328_MOESM6_ESM.docx]

**Supplemental Table 2 Characteristics of the study subjects**

|  | Control group | Postmenopausal osteoporosis patients |
| --- | --- | --- |
| Number | 6 | 6 |
| Age, years | 57.54±8.35 | 60.21±7.88 |
| Height (cm) | 158.77±6.23 | 156.23±5.05 |
| Weight (kg) | 59.23±8.77 | 54.35±8.23 |
| BMI (kg/m^2^) | 24.58±3.57 | 23.57±3.21 |
| Age of menarche (years) | 14.02±2.11 | 14.1±1.92 |
| Age of menopause (years) | 49.15±4.27 | 49.76±4.65 |
| Lumbar spine BMD (g/cm^2^) | 1.23±0.15 | 0.71±0.14* |
| Lumbar spine *T* score | 0.33±1.88 | -2.93±1.21* |
| Total hip BMD (g/cm^2^) | 1.03±0.18 | 0.73±0.17* |
| Total hip *T* score | 0.31±1.68 | -1.72±0.95* |

Data are shown in the form of the mean ± SD, n=6 in each group. P values for all variables are the result of independent t tests between the control and osteoporosis groups, * indicates P < 0.05 compared with the control group.

BMI body mass index, BMD bone mineral density
