# Supplementary material for: TRAF4 positively regulates the osteogenic differentiation of mesenchymal stem cells by acting as an E3 ubiquitin ligase to degrade Smurf2
Source: Cell Death Differ. 2019 May 10;26(12):2652–66. doi: 10.1038/s41418-019-0328-3 (PMC7224386; doi:10.1038/s41418-019-0328-3)
Supplement: Supplementary file 9 — Supplementary figure legends [file 41418_2019_328_MOESM9_ESM.docx]

**SUPPLEMENTARY FIGURE LEGENDS**

**Supplemental Figure 1.** (A) Sh-TRAF4 significantly decreased the protein level of TRAF4 in MSCs, while OE-TRAF4 caused overexpression of the protein level of TRAF4 in MSCs. (B) Neither the decrease nor overexpression of TRAF4 in MSCs affected the growth curve of MSCs in the osteogenic medium. All data are presented as the means ± SD. *p < 0.05 (n=3 independent experiments with 3 different MSC lines).

**Supplemental Figure 2.** After osteogenic induction of MSCs for 14 days, the osteogenic capacity of MSCs in different groups was evaluated. (A) TRAF4 knockdown by sh-TRAF4 decreased ARS and ALP staining (scale bar = 250 µm), while the simultaneous expression of the mutant TRAF4 could rescue the ARS and ALP staining compared with that observed in the sh-TRAF4 group (**black arrows**). (B) Smurf2 knockdown by sh-Smurf2 increased ARS and ALP staining (scale bar = 250 µm), while the simultaneous expression of the mutant Smurf2 could decrease ARS and ALP staining compared with that observed in the sh-Smurf2 group (**black arrows**). (C) TRAF4 knockdown decreased the expression of Runx2 and OCN, while the simultaneous expression of the mutant TRAF4 increased the expression of Runx2 and OCN compared with that observed in the sh-TRAF4 group. (D) Smurf2 knockdown increased the expression of Runx2 and OCN proteins, while the simultaneous expression of the mutant Smurf2 decreased the expression of Runx2 and OCN compared with that observed in the sh-Smurf2 group. All of the data are presented as the means ± SD. *p < 0.05 (n=3 independent experiments with 3 different MSC lines).

**Supplemental Figure 3.** (A) Flag-Smurf2 and Myc-TRAF4 were cotransfected

into 239T cells. At 36 h after transfection, cell lysates were harvested, and Myc-TRAF4 or Flag-Smurf2 was immunoprecipitated with anti-Myc or anti-Flag. Western blotting demonstrated that exogenous Myc-TRAF4 and Flag-Smurf2 interact with each other. (B) Western blotting demonstrated that diminished TRAF4 increased the protein level of Smurf2 in MSCs, while overexpressed TRAF4 decreased the protein level of Smurf2 in MSCs during osteogenesis. Neither the reduction nor overexpression of TRAF4 affected the mRNA level of Smurf2 in MSCs during osteogenesis. (C) TRAF4, Smurf2 and Smurf1 mRNA levels remained unchanged during the osteogenic differentiation of MSCs. (D) The Smurf2 protein level decreased, while that of Smurf1 remained unchanged during the osteogenic differentiation of MSCs. (E) Sh-Smurf2 significantly decreased the protein level of Smurf2 in MSCs, while OE-Smurf2 increased the protein level of Smurf2 in MSCs. Sh-Smurf2 increased the protein levels of Smad1 and Runx2 in MSCs, while the overexpression of Smurf2 decreased the protein levels of Smad1 and Runx2 in MSCs. All data are presented as the means ± SD. *p < 0.05 (n=3 independent experiments).

**Supplemental Figure 4.** (A) Immunofluorescence staining (Scale bar = 50 µm) demonstrated that Smad1 expression was lower in the OCN^+^ osteoblast lineage in OVX rats than in sham-operated rats (**white arrows**). (B) Immunofluorescence staining (scale bar = 50 µm) demonstrated that the expression of Smad1 was lower in the OCN^+^ osteoblast lineage in osteoporotic patients than in control patients (**white arrows**).

(C) Immunofluorescence staining (Scale bar = 50 µm) demonstrated that Runx2 expression was lower in the OCN^+^ osteoblast lineage in OVX rats than in sham-operated rats (**white arrows**). (D) Immunofluorescence staining (scale bar = 50 µm) demonstrated that the expression of Runx2 was lower in the OCN^+^ osteoblast lineage in osteoporotic patients than in the control patients (**white arrows**).
